# Associations of Individual-Related and Job-Related Risk Factors with Nonfatal Occupational Injury in the Coal Workers of Shanxi Province: A Cross-Sectional Study

**DOI:** 10.1371/journal.pone.0134367

**Published:** 2015-07-31

**Authors:** Yan Cui, Shuang-Shuang Tian, Nan Qiao, Cong Wang, Tong Wang, Jian-Jun Huang, Chen-Ming Sun, Jie Liang, Xiao-Meng Liu

**Affiliations:** 1 Department of Health Statistics, School of Public Health, Shanxi Medical University, Taiyuan, China; 2 Department of Health Statistics, Taiyuan Xinghualing District Food & Drug Administration, Taiyuan, China; 3 Department of Neurosurgery, General Hospital of Datong Coal Mining Group, Taiyuan, China; 4 Department of Urology, General Hospital of Datong Coal Mining Group, Taiyuan, China; 5 Center for Postgraduate Education Innovation of Shanxi Province, Taiyuan, China; University of New South Wales, AUSTRALIA

## Abstract

**Objectives:**

To assess the relationships between the risk factors and the incidence of nonfatal occupational injury of coal mine workers of Shanxi Province.

**Methods:**

A cross-sectional study was conducted from July 2013 to December 2013, and 4319 workers were recruited from more than 200,000 coal mine employees who are exposed to continuous potential risk of occupational injuries by using a two-stage stratified cluster sampling method. Trained interviewers having necessary medical knowledge conducted face-to-face interviews with the participants. Univariate and multivariable logistic regression models were used to estimate the odds ratio (OR) and the 95% confidence interval (CI).

**Results:**

A total number of 3618 effective respondents were got from 4319 participants (83.77%) and the mean age of the participants was 41.5 years with the standard deviation of 8.65. Significant crude odds ratios were observed for all factors considered except for marital status, education, work duration, BMI, EPQ-RSC(P) scale and EPQ-RSC(L) scale. Results from multivariable logistic regression model showed significant adjusted odds ratios for risk factors including gender (female *vs* male 0.275, 0.094–0.800), age (≥55 *vs* ≤25yr 0.169, 0.032–0.900), work type (light physical labor *vs* heavy physical labor 0.504, 0.328–0.774), workplace (underground auxiliary *vs* underground front-line 0.595, 0.385–0.919), length of shiftwork experience (0~5yr *vs* no shift 2.075, 1.287–3.344 and ≥15yr *vs* no shift 2.076, 1.230–3.504) and EPQ-RSC(E) score (extraversion *vs* introversion 0.538, 0.334–0.867).

**Conclusions:**

Several risk factors of nonfatal occupational injury were identified including male, age, heavy physical labor, underground front-line, length of shiftwork experience and introversion. The coal mining enterprises should pay attention to controlling the hazards associated with frontline physical work. Workers’ behaviors, life styles and personality traits should also be considered, so that the enterprises could set achievable targets for workers and lessen the exposed period to the risky underground workstation.

## Introduction

An occupational injury to a mine worker often occurs at a mine and for which medical treatment is performed, or which results in death or loss of consciousness, restriction of work or motion, inability to perform all job duties on any day after that, lost workdays, temporary assignment to other duties, transfer to another job, or termination [1].

It has been estimated that there are more than 350,000 workplace fatalities and more than 270 million workplace injuries annually worldwide [2]. In 2010, 363,383 various accidents and 79,552 fatal injuries occurred in China [3]. As a result, occupational accidents are a major public health problem, especially in developing countries.

The mining industry accounts for a significant proportion of occupational injuries in all industry divisions. Mining, especially coal mining, has been considered one of the world's most dangerous occupations and results in severe socio-economic consequences for workers and society [4]. In order to develop effective preventive measures, information about associated risk factors is required. Over many years, a large number of individual-related, job-related, and equipment-related factors have been found and examined in different studies [5–9].

### Individual-related factors

Age is the most investigated risk factor. Margolis (2010) found that as age increases the number of days away from work following an injury also increases. However, Mitchell (1988) and Chau et al. (2014) have shown that employees under the age of 25 rather than older ones are more likely to be injured [6, 10–12]. Living habits (smoking and drinking) are observed to be significantly associated with injury events. Workers who were regular consuming alcohol had OR 2.46 times higher compared to other workers (Kunar et al., 2008) [4, 13–15]. Looking at the impact that obesity has on injury rates, Kouvonen et al. (2013) found that obesity was associated with a higher overall risk of occupational injury [16]. Nakata et al.’s (2005) study in Japanese Small and Medium-scale Enterprises showed that poor nocturnal sleep habit was related to a significantly higher prevalence of injury. Salminen et al. (2010) reported that sleep disturbances (difficulty in initiating sleep, difficulty in maintaining sleep and non-refreshing sleep) increased the occurrence of occupational injury [17, 18]. Education has also been found to have an association with occupational injury, such that no formal education was associated with markedly high risks of injuries [11, 14]. Some psychological traits (extraversion, emotional instability and negative affectivity) have been associated with a strong increase in the risk of injury [19–21].

### Job-related factors

The commonest job-related factors found by the researchers are: work type, workplace, work duration, length of shiftwork experience, job burnout and job dissatisfaction. Workers new to the job are at a much higher risk of injury than more experienced staff, while shift workers and heavy physical workers also have a greater risk of being injured at work [6, 7, 12, 20, 22–24]. A higher risk was found for workers with job burnout and job dissatisfaction [13, 25–29].

### Equipment-related factors

It has been reported that working as a facilities or machine operator or assembler, poor workplace conditions and undesirable work environment were risk factors for occupational injury [5]. Groves et al. used Mine Safety and Health Administration (MSHA) and Current Population Survey (CPS) data to examine equipment-related injuries over the period 1995–2004 [8]. The results showed that 37%-88% of the total mine fatalities were attributable to equipment each year, and non-powered hand tools was the most frequently involved equipment category with nonfatal injuries while off-road ore haulage was the most common source of fatalities. Moreover, despite many ongoing safety initiatives around the world, working in an underground coal mine is rarely as safe as working in an office [30, 31].

Most researches on occupational injury of coal mining enterprises in China have focused on death and serious injury. However, nonfatal injury accounted for the majority in the occupational harm. Some surveys showed that the proportion of minor injury, serious injury, and death was 350: 23: 1 [24, 32].

Therefore, this study aimed to assess the relationships of risk factors, including gender, age, education, marital status, monthly income, living habits, BMI, work type, workplace, length of shiftwork experience, work duration, job satisfaction, job burnout and EPQ-RSC, with the incidence of nonfatal occupational injury in coal mine workers from a large-scale coal enterprise in northern Shanxi Province. Logistic regression was used in the cross-sectional study to compute OR and 95% CI. The results will help to reduce the occurrence of occupational injuries by suggesting some potential prevention and control measures.

## Materials and Methods

This cross-sectional study was conducted on workers from a large coal mine group located in the north of Shanxi Province, with the coal field covering 6157 square kilometers and the total coal reserves being 89.2 billion tons. The management of the coal mine group provided us the baseline data which contains gender, date of birth, work type for the development of the sampling frame. According to the targets, the study used a two-stage cluster sampling method. In the first stage, we randomly sampled 10 coal mines from 87 coal mines of three coal group areas (Pingwang Region, Kouquan Trench, Yungang Trench) as the primary sampling unit (PSU). In the second stage, a stratified random sampling method was applied to select participants by gender, age and the type of work. Considering the acceptable absolute difference of 0.01 between the sample and the population prevalence, αtype I error of 0.05, and confidence interval (1-α) of 0.95, a sample size of 4154 deliveries will be necessary, according to the following formula:

$$\begin{array}{l} n=(\frac{U^{2}\pi (1-\pi )}{\delta^{2}})\\ n_{c}=\frac{n}{1+n/N} \end{array}$$

$$n_{c}=4154$$

Where, *U* is the two-tailed standard normal variate value related to the null hypothesis, and *π* is the injury rate, *δ* is allowable error. *N* is the population of the coal mine group.

Taking into considerations on the potential of lost to follow up and withdraw from the study, we aimed to survey 4400 coal miners.

A pilot study was implemented for a feasibility analysis. With respect to the formal research, the interviews were processed 5 days a week and about 300 participants were investigated each week. The management of the organization contacted the involved workers two days before the interview and made a rough survey time schedule. Eight trained interviewers having necessary medical knowledge conducted face-to-face interviews with the participants. The interview for each person required a time span of 40 min in their workplace.

The survey used an anonymous questionnaire based on previous relatedliterature [20, 24, 33]. It comprised three components: (1) general information: gender, date of birth, weight, height, educational level, marital status, work type, workplace, work duration (current employment), length of shiftwork experience (the mines operate seven days a week and three shifts per day for coal production), work dangerousness (self-reported), smoking status (current smokers and non-smokers), drinking status, sleep status (an assessment of usual sleep quality). (2) occupational injury information: the injury workplace, type of injury, localization of lesions and the severity of injury. (3) psychological scales: Revised Eysenck Personality Questionnaire Short Scale for Chinese (EPQ-REC), MBI-GS job burnout scale.

### Participants

The survey was conducted from July 2013 to December 2013, and 4319 workers were recruited from the population of more than 200,000 employees in this coal company who are exposed to continuous potential risk of occupational injuries. The study was approved by Shanxi Medical University Ethics Committee with the following statement: The design of this study accorded with the ethical requirements, and agreed to declare. Eligible participants who were permanent staff of these mines, aged between 18 and 65 yr, had previously been informed of the objectives of the study, and had given their written informed consent.

### Identification of occupational injury

Retrospecting the past 5-year period, the injury workplace, type of injury, localization of lesions and the severity of injury were reported by the surveyed workers.

Type and localization of the injury were identified by the "Classification criteria for enterprise workers casualty" (GB 6441–86). The questionnaire includes 7 types of injuries (smashing injury, blast injury, mechanical traffic injury, falling injury, sprains and luxation, poisoning and others) and 5 localizations of lesions (head and face, trunk, limbs, whole body and others) [34].

The severity of injury took the "Identification criteria for occupational injury and occupational disability" (GB / T16180-2006) as a reference. The degree of injury is divided into three categories, namely (1) minor: recovery or rehabilitation; (2) moderate: activity limitation or discomfort; (3) serious: disabled [35]. We eliminated the participants who had occupational diseases, got injury from others, or died due to work-related accidents.

### Quality control

Strict quality control (QC) was applied to assure the quality of data collection. Firstly, a QC team was established to develop an investigator training materials including definition of eligible participants, study population, and sampling procedure. To control the recall errors, two of the authors tried to verify the injury history which was registered in the coal mine hospital. And the concordance rate was 95.65% (6 minor injury workers didn’t have injury history). Moreover, participants who reported no injury also had no record in the mine hospital. All investigators attended a one-week training course and passed the course evaluation before appointment. Secondly, in order to assure the response rate, we informed the eligible participants via management of the coal mines and conducted follow-up visits. If the eligible participants could not take part in the survey at a scheduled time, the QC team tracked and followed up with them until they were able to participate in the survey.

### Statistical analysis

Data were double-entered into Epi info version 3.5.1 (CDC, Atlanta, USA) which reduces error in creating electronic dataset prior to statistical analysis. Age was generated from the birth date. All independent variables were categorized (see S1 table) and described with frequency distribution. Chinese Body Mass Index (BMI) criteria (normal: BMI< = 23.9, overweight: 24 = <BMI< = 26.9, obesity: BMI> = 27) was used to classify BMI. Overweight and obesity were classified together into a group in the analysis. The EPQ-REC original score was transformed into a T score according to Chinese norms, and then categorized into three groups (<43.3, 43.3–56.7 and >56.7). Job burnout total score was also categorized into three groups(<50, 50–75 and ≥75). The outcome variable considered in the model was categorized as a dichotomous variable (injury or no-injury). To access the relationships between various factors with injury, the crude odds ratios (OR) and their 95% confidence intervals (CI) were calculated with univariate logistic regression. The adjusted odds ratios (ORa) were then estimated using multivariable logistic regression with stepwise procedure. All these statistical analysis were conducted using SAS version 9.3 (SAS Institute, Inc., Cary, NC, USA), and a level of 0.05 was used to declare statistical significance.

## Results

A total number of 3618 effective respondents were got from the 4319 participants who had been recruited (83.77%). The mean age of coal miners was 41.5 years with standard deviation 8.65. The median time that passed between each participant's last injury and their interview was 1.54 years. Table 1 shows that there were 137 reported accidents among the 3618 coal workers (3.79%) who experienced at least one accident. The total number of injuries was 138: with one accident n = 136 (3.76%); with two accidents n = 1 (0.03%).

**Table 1:** Distribution of the nonfatal occupational injures (%).

The number of injuries	Frequencies (n)	Proportion (%)	Prevalence (%)
0	3481	96.21	
1	136	3.76	3.76
2	1	0.03	0.03
Total	3618	100.0	

Table 2 presents the distribution of injuries according to workplace, type of injury, localization of injury and severity of injury. For all injuries, underground represented about 79.71%, and above ground about 20.29%. Smashing injury was the most common injury accounting for 53.63%, sprains and luxation 23.92%. The localization of injury happened mainly in limbs (57.25%), followed by head and face (18.12%) and trunk (17.39%). The majority of injuries (60.87%) were minor injury.

**Table 2:** Characteristic distribution of nonfatal occupational injuries.

Variable	Characteristics	n	Proportion (%)
Type of injury	smashing injury	74	53.63
	blast injury	5	3.62
	mechanical traffic injury	9	6.52
	falling injury	5	3.62
	sprains and luxation	33	23.92
	poisoning	3	2.17
	others	9	6.52
Localization of injury	head and face	25	18.12
	trunk	24	17.39
	limbs	79	57.25
	whole body	3	2.17
	others	7	5.07
Severity of injury	minor	84	60.87
	moderate	50	36.23
	serious	4	2.90

Table 3 indicates that workplace dangerousness had significant differences in different age groups. Workers aged 55 or more tended to rate their workplace as never dangerous, more often than younger workers. Significant differences also existed between males and females. 68.79% females rated their workplace as never dangerous, but for males the value was only 20.90%.

**Table 3:** The danger of the work environment in different groups.

		The danger of the work environment (%)					*P*
		Never	Seldom	Sometimes	Often	Usually	
**Age**	≤25yr	24.72	19.10	26.97	13.48	15.73	0.008
	25~35yr	26.65	15.53	24.70	14.13	18.99	
	35~45yr	27.50	17.58	25.09	11.12	18.71	
	45~55yr	29.37	17.78	25.70	11.50	15.65	
	≥55yr	40.25	20.34	20.76	10.17	8.47	
**Gender**	male	20.90	17.58	27.98	13.53	20.01	<0.001
	female	68.79	16.03	8.97	3.97	2.24	

Table 4 observes that 79.59% males performed physical labor, and females 45.68%. Current smoking and drinking status had significant differences between physical labor and mental labor groups.

**Table 4:** Comparison of gender, smoking and drinking status between two groups.

		Work type		*P*
		Physical labor N (%)	Mental labor N (%)	
**Gender**	male	2418(79.59)	620(20.41)	<0.001
	female	266(45.86)	314(54.14)	
**Smoking**	yes	1686(81.73)	377(18.27)	<0.001
	no	998(64.18)	557(35.82)	
**Drinking**	yes	1127(77.40)	329(22.60)	0.003
	no	1557(72.02)	605(27.98)	

### Univariate analysis

To start with, all suggested factors were investigated as possible independent variables by fitting the univariate logistic regression model in which the dependent variable is dichotomous, notably presence or absence of an injury.

The distribution of injury showed that among those workers who gave history of injuries, male represented about 97.08% (133), female 2.92% (4). The majority of the injured were between 25 to 45 years old (72.27%). About 91.24% of the injured were married, and 64.23% completed junior college or senior high school.

Table 5 shows that significant crude odds ratios (OR) were observed for all factors considered except for marital status, education, work duration, BMI, EPQ-RSC(P) scale and EPQ-RSC(L) scale. Male, age, heavy physical work, underground front-line, length of shiftwork experience, monthly income (6,000–8,000RMB), average or bad sleep, smoking, drinking, job burnout, job dissatisfaction, introversion and emotional instability had significant associations with a higher risk of occupational injury.

**Table 5:** Univariate analysis of logistic regression.

Factor		Injury (N(%))		*χ* ^*2*^	*P*	*OR*	95%*CI*
		yes(137)	no (3481)				
Gender	male	133(4.38)	2905(95.62)	13.70			
	female	4(0.69)	576(99.31)		<0.001**	0.152	0.056–0.412
Age	≤25yr	6(6.74)	83(93.26)	13.34			
	25~35yr	48(5.18)	879(94.82)		0.531	0.755	0.314–1.818
	35~45yr	51(3.83)	1280(96.17)		0.182	0.551	0.230–1.322
	45~55yr	30(2.90)	1005(97.10)		0.055	0.413	0.167–1.020
	≥55yr	2(0.85)	234(99.15)		0.010**	0.118	0.023–0.597
Marital status	married	125(3.77)	3189(96.23)	0.0235			
	single	12(3.95)	292(96.05)		0.878	1.048	0.573–1.919
Educational level	bachelor degree or above	12(2.65)	440(97.35)	1.84			
	junior college and senior high school	88(4.00)	2114(96.00)		0.176	1.526	0.828–2.814
	junior high school or below	37(3.84)	927(96.16)		0.259	1.464	0.756–2.834
Work type	heavy physical	72(7.78)	854(92.22)	49.52			
	light physical	48(2.73)	1710(97.27)		<0.001**	0.333	0.229–0.484
	mental labor	17(1.82)	917(98.18)		<0.001**	0.220	0.129–0.376
Workplace	underground	64(8.50)	689(91.50)	55.48			
	underground auxiliary	45(3.59)	1207(96.41)		<0.001**	0.401	0.271–0.594
	ground	21(2.32)	884(97.68)		<0.001**	0.256	0.155–0.423
	office	7(0.99)	701(99.01)		<0.001**	0.108	0.049–0.236
Work duration	≤1yr	8(5.26)	144(94.74)	2.71			
	2~10yr	49(4.11)	1143(95.89)		0.508	0.772	0.358–1.662
	11~20yr	42(3.95)	1021(96.05)		0.448	0.740	0.341–1.609
	≥21yr	38(3.14)	1173(96.86)		0.176	0.583	0.267–1.274
Length of shiftwork experience	no shift	39(2.02)	1893(97.98)	34.65			
	0~5yr	41(6.71)	570(93.29)		<0.001**	3.492	2.230–5.466
	5~15yr	24(4.91)	465(95.09)		0.0005**	2.505	1.492–4.207
	≥15yr	33(5.63)	553(94.37)		<0.001**	2.896	1.805–4.649
Monthly income	≤4,000	26(2.78)	909(97.22)	6.46			
	4,000~6,000	55(3.61)	1469(96.39)		0.265	1.309	0.815–2.102
	6,000~8,000	41(5.06)	770(94.94)		0.015*	1.862	1.128–3.071
	≥8,000	15(4.31)	333(95.69)		0.169	1.575	0.824–3.010
Sleep status	good	54(2.85)	1842(97.15)	10.22			
	average	69(4.65)	1415(95.35)		0.006**	1.663	1.157–2.391
	bad	14(5.88)	224(94.12)		0.014*	2.132	1.165–3.900
Smoking	no	46(2.96)	1059(97.04)	5.07			
	yes	91(4.41)	1972(95.59)		0.024*	1.514	1.055–2.172
Drinking	no	69(3.19)	2093(96.81)	5.17			
	yes	68(4.67)	1388(95.33)		0.023*	1.486	1.056–2.092
BMI	normal	52(3.42)	1467(96.58)	0.946			
	overweight or obesity	85(4.05)	2014(95.95)		0.331	1.191	0.838–1.692
Job burnout	normal	81(3.24)	2416(96.76)	8.62			
	slight burnout	53(4.85)	1040(95.15)		0.020*	1.520	1.067–2.165
	burnout	3(10.71)	25(89.29)		0.040*	3.579	1.059–12.098
Job satisfaction	satisfied	80(3.22)	2408(96.78)	8.94			
	moderate	49(4.78)	976(95.22)		0.026*	1.511	1.051–2.173
	dissatisfied	8(7.62)	97(92.38)		0.018*	2.484	1.168–5.282
EPQ-RSC(E)	introversion	35(5.43)	609(94.57)	6.70			
	middle	62(3.74)	1596(96.26)		0.071	0.676	0.442–1.034
	extraversion	40(3.04)	1276(96.96)		0.010**	0.545	0.343–0.867
EPQ-RSC(P)	mild	37(3.61)	987(96.39)	0.42			
	middle	82(3.76)	2096(96.24)		0.834	1.044	0.703–1.550
	obstinate	18(4.33)	398(95.67)		0.522	1.206	0.679–2.144
EPQ-RSC(N)	emotional stability	35(2.96)	1147(97.04)	4.99			
	middle	54(3.76)	1383(96.24)		0.264	1.280	0.830–1.972
	emotional instability	48(4.80)	951(95.20)		0.026*	1.654	1.061–2.579
EPQ-RSC(L)	low masked	17(4.67)	347(95.33)	2.29			
	middle	65(4.10)	1521(95.90)		0.624	0.872	0.505–1.506
	high masked	55(3.30)	1613(96.70)		0.201	0.696	0.399–1.213

** *P* ≤0.01,

* 0.01<*P* ≤0.05;

*OR* = odds Ratio

### Multivariable analysis

Based on the results from the univariate logistic regression models, we perform a multivariable logistic regression analysis by using the stepwise method (criterion for entry = 0.05 and retention = 0.1) with all the variables that could be selected as suggested injury risk factors (univariate p< 0.05). Variance inflation factors (VIF) was used to detect collinearity with the SAS command PROC REG, and the result indicated that there was no evidence of multicollinearity as all VIF values were less than 10. As noted in Table 6, significant adjusted odds ratios were found for gender (female *vs* male 0.275, 0.094–0.800), age (≥55 *vs* ≤25yr 0.169, 0.032–0.900), work type (light physical labor *vs* heavy physical labor 0.504, 0.328–0.774), workplace (underground auxiliary *vs* underground front-line 0.595, 0.385–0.919), length of shiftwork experience (0~5yr *vs* no shift 2.075, 1.287–3.344 and ≥15yr *vs* no shift 2.076, 1.230–3.504). EPQ-RSC(E) score showed that introversion had a higher risk of injury than extraversion (extraversion *vs* introversion 0.538, 0.334–0.867).

**Table 6:** Multivariable analysis of logistic regression.

	$\hat{\beta }$	*P*	*OR*	95%*CI*
**Gender**				
female *vs* male	-1.292	0.018*	0.275	0.094–0.800
**Age**				
25~35yr *vs* ≤25yr	-0.015	0.975	0.985	0.394–2.467
35~45yr *vs* ≤25yr	-0.463	0.334	0.629	0.246–1.610
45~55yr *vs* ≤25yr	-0.689	0.169	0.503	0.189–1.340
≥55yr *vs* ≤25yr	-1.778	0.037*	0.169	0.032–0.900
**Type**				
light physical labor *vs* heavy physical labor	-0.685	0.002**	0.504	0.328–0.774
mental labor *vs* heavy physical labor	-0.606	0.067	0.545	0.285–1.043
**Work place**				
underground auxiliary *vs* underground front-line	-0.520	0.019*	0.595	0.385–0.919
ground *vs* underground front-line	-0.478	0.105	0.620	0.348–1.105
office *vs* underground front-line	-0.952	0.054	0.386	0.147–1.015
**Length of shiftwork experience**				
0~5yr *vs* no shift	0.730	0.003**	2.075	1.287–3.344
5~15yr *vs* no shift	0.455	0.102	1.576	0.914–2.716
≥15yr *vs* no shift	0.730	0.006**	2.076	1.230–3.504
**EPQ-RSC (E)**				
middle *vs* introversion	-0.382	0.086	0.683	0.442–1.055
extraversion *vs* introversion	-0.619	0.011*	0.538	0.334–0.867

** *P* ≤0.01,

* 0.01<*P* ≤0.05;

*OR* = odds Ratio.

## Discussion

This cross-sectional study demonstrates that male, heavy physical labor, underground front-line, length of shiftwork experience (0~5yr, ≥15yr) and introversion were associated with markedly higher risks of nonfatal occupational injury. Older workers were less likely to be injured rather than those under the age of 25. It should be noted that the median time that passed between each participant's last injury and their interview was 1.54 years. Time interval is a major determinant to recall injury accuracy and the longer the recall period, the more recall errors will be [36, 37]. Verifications of the self-reported injuries against records in the mine hospital were used to control the problem of recall bias. A two-stage stratified cluster sampling method (complex sampling design) which combines the simplicity of cluster sampling and high estimation accuracy of stratified sampling was used to select participants. Face-to-face interviews were conducted so that participants did not face any difficulties in responding the questionnaire items.

The distribution of injuries according to workplace indicated that 79.71% of the accidents were found to occur in the underground. Smashing injury (55.1%) and sprains (23.92%) were the most common injuries, and the limbs were the most vulnerable localization of injury (57.25%). These characteristics distributions were consistent with the nonfatal occupational injury of mining industries [2]. Similar proportions of minor versus more serious injuries have been consistently observed from the previous study [24].

Incidence rates reported by the Bureau of Labor Statistics consistently show lower injury risk for female compared with male [38], and our study shows the same results. The increased risk of occupational injury for male was probably a reflection of the different jobs males and females performed within the study coal groups (Table 4). Table 3 showed that men faced a more dangerous work environment than women did and thus had higher probability of being injured.

The present study reports that older workers were less likely to be injured than younger workers. Table 3 presented that 40.25% of the workers in the ≥55yr group were placed in non-hazardous jobs. There are several possible explanations for this result. Older workers possess lower physical work capacity due to a decrease in aerobic and musculoskeletal capacity [6, 39]. Considering this, the management might have changed the older workers’ occupations into a relatively safe one. In addition, increasing age brings about more experience and familiarity with the work environment, so that older workers might possess a compensatory ability to reduce difficulties and avoid injury in meeting job demands [12]. Besides, younger workers are associated with lack of knowledge, inexperience and risk behaviors [10, 12].

Both univariate and multivariable logistic regression reveal that workplace had a significant influence on injury occurrence. The front-line underground workers represented a higher risk of occupational injury than those worked in underground auxiliary, ground and office, which was a common feature of mining enterprises. There are more chances of getting injured in underground due to more hazards. Underground workers are more specifically exposed to manual material handling and machine-related hazards that greatly lead to occurrence of occupational injury [14]. Workplace factors like exposed roof, slippery floor, heat and space availability have also been found to be responsible in causing injuries in construction industry [14, 22]. This study also reports that heavy physical labor played a role in occupational injury, and the result could be explained by the fact that 87.37%of the heavy physical labor is located in underground.

A marked risk was found for workers with length of shiftwork experience. The study presents that length of shiftwork experience less than 5years or more than 15 years had an increased risk of injury compared to no-shift workers. With respect to the individual aspects, one of the main detrimental effects of shiftwork is the disturbance of the normal circadian rhythm of the psychophysiological functions, which lead to fatigue and increase the risk of injury [40, 41]. This disturbance tends to be associated with sleep disorder that can result in distractibility, nervousness, irritability and anxiety [40, 42]. However, workers with mid-range shiftwork experience (5–15 years) were not significant compared to non-shift workers. The mid-range workers may have better tolerance to shiftwork [41].

Another important finding in our study is that introversion was a significant predictor of injury. Several studies suggested that extraversion, marked by overconfidence, intolerance, and aggression, was more prone to accidents due to risk taking behaviors and carelessness [19, 22, 24, 43]. However, our result was exception, rather than trend. Marusic et al. (2001) proposed that introverted participants felt significantly more responsible for the sustained injuries than extraverts [44]. This result has to be made with caution and need further discussion.

Job burnout and job dissatisfaction along with emotional instability were found to be associated with higher injury risk in univariate analysis but not in multivariable analysis. Thesefactorsthat influencemutually may lead to higher levels of distractibility, thereby increasing the risk of injury [20–22].

Lifestyle parameters related to injury are smoking and drinking. The results of logistic regression indicates that smoking and drinking had strong significant crude odds ratios (OR 1.514, 1.486 respectively), but non-significant adjusted odds ratios. The result of chi-square test (Table 4) provided significant differences between physical labor and mental labor for the current smoking and drinking status. Current smoking and drinking status are often correlated with the blue collar occupations which are more hazardous.

Univariate analysis result shows that monthly income of 6000–8000 RMB was a risk factor for the occupational injury, which was different from the related studies that workers with high monthly income had lower risk of injuries [32, 45]. This finding was expected as workers in this income range, mostly worked in underground front-line, always in more dangerous working conditions.

Initially it was suspected that education, work duration and BMI were associated with the occupational injury, but our results obtained with the logistic regression model have presented that they had no significant effect on occupational injury. One possible explanation is that there are significant sociocultural differences between participants investigated and those of the other studies.

## Conclusions

Several risk factors of nonfatal occupational injury were identified including male, age, heavy physical labor, underground front-line, length of shiftwork experience and introversion. The coal mining enterprises should pay attention to controlling the hazards associated with frontline physical work. Workers’ behaviors, life styles and personality traits should also be considered, so that the enterprises could set achievable targets for workers and lessen the exposed period to the risky underground workstation.

## Supporting Information

S1 Dataset All variables dataset and injury dataset. (XLS)

S1 table Variable assignment table. (DOC)
