# Supplementary material for: Associations of Individual-Related and Job-Related Risk Factors with Nonfatal Occupational Injury in the Coal Workers of Shanxi Province: A Cross-Sectional Study
Source: PLoS One. 2015 Jul 31;10(7):e0134367. doi: 10.1371/journal.pone.0134367 (PMC4521807; doi:10.1371/journal.pone.0134367)
Supplement: S1 table — (DOC) [file pone.0134367.s002.doc]

**S1 Table. Variable assignment t**able

| Variable | Assignment |
| --- | --- |
| Gender | male=1, female=2 |
| Age | ≤25yr=1, 25~35yr=2, 35~45yr=3, 45~55yr=4, ≥55 yr=5 |
| Marital status | married=1, single=2, |
| Educational level | bachelor degree or above =1, junior college and senior high school =2, junior high school or below=3 |
| Work type | heavy physical=1, light physical=2, mental labor=3 |
| Workplace | underground=1, underground auxiliary=2, ground=3, office=4 |
| Work duration | ≤1 yr=1, 2~10 yr=2, 11~20yr=3, ≥21yr=4 |
| Length of shiftwork experience | no shift=1, 0~5yr=2, 5~15 yr=3, ≥15 yr=4 |
| Work dangerousness | never=1, seldom=2, sometimes=3, often=4, usually=5 |
| Monthly income | ≤4000yuan=1, 4000-6000yuan=2, 6000-8000yuan=3, ≥8000yuan=4 |
| Sleep status | good=1, average=2, bad=3 |
| Smoking * | no = 0 , yes=1 |
| Drinking | no = 0 , yes=1 |
| BMI* | normal=1, overweight=2 , obesity =3 |
| Job burnout | normal =1, slight burnout= 2, burnout =3 |
| Job satisfaction | satisfied =1, moderate= 2, dissatisfied =3 |
| EPQ-RSC (E) | introversion =1, middle =2, extraversion= 3 |
| EPQ-RSC (P) | mild =1, middle= 2, obstinate= 3 |
| EPQ-RSC (N) | emotional stability =1, middle= 2, emotional instability =3 |
| EPQ-RSC ( L) | low masked =1, middle =2, high masked = 3 |
| Type of injury | smashing injury=1, blast injury=2, mechanical traffic injury=3, falling injury=4, sprains and luxation=5, poisoning=6, others=7 |
| Localization of injury | head and face=1, trunk=2, limbs=3, whole body=4, others=5 |
| Severity of injury | minor=1, moderate=2, serious=3 |

* ‘Yes’ means ‘current smokers’ – Adults who have smoked 100 cigarettes in their lifetime and currently smoke cigarettes every day (daily) or some days (nondaily).

‘No’ means ‘non-smokers’– Adults who have never smoked a cigarette or who smoked fewer than 100 cigarettes in their entire lifetime.

In our study, we classify the participants who have smoked more than 100 cigarettes but who no longer smoke at all into ‘non-smokers’ group.

*BMI– overweight and obesity were classified together into a group in analysis.
